# A novel virtual reality-integrated multi-modal intervention for community-dwelling older adults with mild cognitive impairment: protocol for a randomized controlled trial

**DOI:** 10.3389/fnagi.2026.1721346

**Published:** 2026-03-05

**Authors:** Yuanbao Sun, Zhuorong Zi, Songli Wang, Minghui Weng, Jin Peng, Linwan Liu, Jian Zhou, Nianjie Wang, Bo Liu, Kezhi Liu, Youguo Tan

**Affiliations:** 1 School of Psychiatry, North Sichuan Medical College, Nanchong, China; 2 Department of Psychiatry, Zigong Mental Health Center, Zigong, China; 3 Research Center for Psychiatry, Zigong Institute of Brain Science, Zigong, China; 4 The Affiliated Hospital, Psychosomatic Medicine Center, Southwest Medical University, Luzhou, China

**Keywords:** brain-gut axis, cognitive training, mild cognitive impairment, gut microbiota, physical exercise, virtual reality

## Abstract

**Background:**

Emerging research suggests virtual reality (VR) techniques hold promise for mitigating cognitive decline in patients with mild cognitive impairment (MCI). Furthermore, accumulating evidence indicates that gut dysbiosis is a key factor associated with cognitive impairment. This study aims to determine whether a novel virtual reality-integrated multi-modal intervention can beneficially modulate the brain-gut axis in individuals with MCI.

**Methods:**

This study is a randomized single-blind controlled trial that will include 66 older adults with MCI from the community. Eligible participants will be randomly assigned in a 1:1 ratio to the intervention group or the waitlist group. The intervention group will complete 36 sessions (three sessions per week for 12 weeks) consisting of virtual reality cognitive training (VRCT), traditional cognitive training (TCT), and physical exercise (PE). The control group will not receive any intervention during the study period. The primary outcome is the change in a memory-weighted cognitive composite score. Exploratory outcomes: mechanistic changes along the brain-gut axis, including: (1) Changes in gut microbiota alpha/beta diversity and composition assessed by 16S rRNA gene sequencing, (2) Changes in resting-state brain activity and functional connectivity assessed by fMRI. Outcome measures will be assessed at three or four time points: baseline, mid-intervention (Week 6), post-intervention (Week 12), and at a 12-week follow-up (Week 24).

**Expected outcomes:**

We hypothesize that, relative to the waitlist control, the intervention group will demonstrate concurrent improvements in cognitive performance and a shift in gut microbiota composition toward a more favorable profile, thereby providing preliminary evidence for modulation of the brain-gut axis.

**Clinical trial registration:**

[www.chictr.org.cn], identifier [ChiCTR2400093397].

## 1 Introduction

Mild cognitive impairment (MCI) is an intermediate stage between normal aging and dementia, affecting roughly 10%–15% of adults aged 65 and older (Anderson, 2019). In some cases, MCI can progress to different forms of dementia, such as Alzheimer’s disease (AD) or vascular dementia (VD) (Meyer et al., 2002). With current dementia treatments demonstrating limited effectiveness, researchers are increasingly focusing on early intervention during the MCI stage or even earlier. While pharmacological options exist, non-pharmacological interventions are currently considered the preferred approach for managing MCI (Talar et al., 2022).

Previous research has demonstrated that traditional cognitive training (TCT), physical exercise (PE), and lifestyle modifications can slow cognitive decline in individuals with MCI (Fonte et al., 2019; Ornish et al., 2024). A recent randomized controlled trial (RCT) demonstrated that a 20-week combined intervention of aerobic exercise and cognitive training led to significant improvements in Alzheimer’s Disease Cognitive Assessment Scale (ADAS-Cog-13) scores among patients with MCI (Montero-Odasso et al., 2023). Similar cognitive benefits were observed with mind-body exercises, including Tai Chi (Li et al., 2023) and yoga training (Grzenda et al., 2024). A systematic review revealed that both aerobic exercise and resistance training enhance cognitive performance in both healthy community-dwelling older adults and patients with MCI (Law et al., 2020). Another RCT study found that a 20-week lifestyle modification program significantly improved cognitive function in patients with MCI (Ornish et al., 2024). Furthermore, recent advances in non-pharmacological interventions have established computerized cognitive training (CCT) (Li et al., 2022) and virtual reality cognitive training (VRCT) (Zhong et al., 2021) as evidence-based approaches for mitigating cognitive decline in MCI.

Virtual reality (VR) is emerging as a promising and cost-effective tool for cognitive intervention and physical exercise (García-Betances et al., 2015). Its immersive, interactive, and engaging nature makes it more appealing and motivating than traditional methods, attracting growing interest in recent years (García-Betances et al., 2015). A recent systematic review indicated that VRCT is as effective as traditional approaches, while offering greater ecological validity and adaptability in the treatment of MCI (Tortora et al., 2024). A 12-week single-blind RCT study demonstrated that a VR physical and cognitive training intervention significantly improved overall cognition, delayed recall, and instrumental activities of daily living (Liao et al., 2020). Moreover, functional near-infrared spectroscopy (fNIRS) data from the VR group revealed reduced activation in prefrontal regions following the intervention, indicating improved neural efficiency (Liao et al., 2020). In addition, VR-related biomarkers have shown effectiveness in detecting behavioral patterns associated with subtle deficits in instrumental activities of daily living (Park et al., 2024). When combined with MRI-based biomarkers, such as observable structural brain changes, VR may serve as a valuable tool for the early screening of MCI (Park et al., 2024). However, the effect of combining VRCT, TCT, and PE on cognition remains largely unexplored.

Cognitive function in middle-aged and older adults has also been shown to be associated with specific gut microbiota (Aljumaah et al., 2022). This connection suggests that probiotics, prebiotics, and commensal bacteria may help slow or improve cognitive decline (Aljumaah et al., 2022). Li et al. (2019) observed similar alterations in the gut microbiota of individuals with AD and mild MCI. For instance, the genus Escherichia was found to be elevated in both fecal and blood samples from individuals with AD and MCI (Li et al., 2019). Fan et al. (2025) identified 59 key microbial species associated with biomarkers of MCI and AD. Through functional profiling, they revealed microbial pathways involved in energy metabolism and neuroinflammation, highlighting their potential role in mediating the relationship between gut microbiota and brain health (Fan et al., 2025). However, few studies have examined whether cognitive training can modulate the brain-gut axis in MCI.

We hypothesize that a multi-modal intervention, integrating VRCT, TCT, and PE, will improve cognitive performance and beneficially modulate the brain-gut axis in individuals with MCI. To test this hypothesis, we will conduct a randomized, single-blind controlled trial among community-dwelling older adults with MCI. The primary objective of this study is to investigate whether the multi-modal intervention enhances cognitive function in community-dwelling older adults with MCI. The exploratory objective is to characterize the underlying brain-gut axis mechanisms, as assessed by resting-state functional MRI and 16S rRNA gene sequencing of the gut microbiota.

## 2 Methods and analysis

### 2.1 Study design

This single-blind, randomized controlled trial (ChiCTR2400093397, www.chictr.org.cn) employs a two-arm parallel design, consisting of an intervention group receiving a combination of VRCT, TCT, and PE, and a waitlist control group who will receive no training during the research period. As compensation, the waitlist group will be offered the same training after the follow-up assessment. All participants will undergo comprehensive assessments at four time points: baseline (week 0), mid-intervention (week 6), post-intervention (week 12), and a 12-week follow-up (week 24). The trial is designed in accordance with the CONSORT (Consolidated Standards of Reporting Trials) guidelines and will be conducted and reported accordingly.

### 2.2 Participants

The inclusion criteria are as follows: individuals aged between 60 and 80 years who met the International Working Group criteria for MCI (Winblad et al., 2004), Mini-Mental State Examination (MMSE) score of ≥24 (Zhang et al., 2018), Montreal Cognitive Assessment (MoCA) score of ≤26 (Zhang et al., 2018), Patient Health Questionnaire-9 (PHQ-9) score of <5 (Kroenke et al., 2001), and not taking any cognitive-enhancing medications. Exclusion criteria include individuals with major physical illnesses or other psychiatric disorders, those who have difficulty cooperating, or those unwilling to participate in the study. The full list of inclusion and exclusion criteria is presented in Table 1.

**TABLE 1:** Inclusion and exclusion criteria.

Inclusion criteria	Exclusion criteria
1. Right-handed	1. Individuals with major physical or mental illnesses
2. Aged between 60 and 80 years	2. Individuals who are illiterate or unable to comprehend the study content
3. Meets the International Working Group criteria for MCI	3. Individuals with acute exacerbations of cardiovascular, respiratory, or other chronic conditions
4. MMSE ≥ 24	4. History of substance abuse or dependence
5. MOCA score ≤ 26	5. Use of antibiotics or probiotics within 1 month prior to or during the trial
6. PHQ9 < 5	6. Discomfort with VR equipment
7. Not currently taking any cognitive-enhancing medications	7. Severe visual or hearing impairments
–	8. Contraindications to MRI
–	9. Refusal to participate or failure to provide informed consent

MCI, mild cognitive impairment; MMSE, Mini-Mental State Examination; MoCA, Montreal Cognitive Assessment; PHQ9, Patient Health Questionnaire 9; VR, virtual reality; MRI, Magnetic Resonance Imaging.

This study will be conducted in full compliance with the ethical principles of the World Medical Association’s Declaration of Helsinki (2024 revision) for medical research involving human subjects. Ethical approval was obtained from the Ethics Committee of Zigong Mental Health Center (Approval No. 20241102). All participants will be fully informed about the study protocol and will be required to provide written informed consent prior to enrollment.

### 2.3 Randomization and blinding

Participants will be randomly assigned in a 1:1 ratio to either the intervention group or the waitlist group using a computer-generated randomization sequence. To ensure baseline balance, randomization will be stratified by MCI subtype (amnestic vs. non-amnestic), baseline MoCA score (19–22 vs. 23–26), and age (60–65, 66–70, or 71–80 years). This approach ensures allocation concealment and minimizes selection bias. Due to the practical challenges of blinding participants, the study was conducted as a single-blind randomized controlled trial. Group allocation was concealed from partial research personnel—including outcome raters and data analysts—with the exception of the therapists administering the interventions, in order to reduce observer bias. All raters are explicitly prohibited from inquiring about participants’ group assignments, and all participants are instructed not to voluntarily disclose their treatment experiences or subjective impressions to raters.

### 2.4 Interventions

#### 2.4.1 Physical exercise (PE)

The World Health Organization recommends that older adults engage in moderate- to vigorous-intensity physical activity on a weekly basis, as such activities can improve functional capacity and reduce the risk of falls (Bull et al., 2020). In the present study, the intervention group will be instructed to engage in public square dancing, which is popular among middle-aged and older adults in China—three times per week, with each session lasting 30 min, over a 12-week period (totaling 36 sessions). To ensure consistency, all sessions will utilize a fixed set of low-to-moderate intensity, widely-practiced dance routines. These group-based exercise will be led by a trained instructor following a standardized video-audio recording that dictates the movements, tempo, and duration. All participants will be required to wear a wrist-strap heart rate monitor during exercise, with intensity maintained at a moderate level—defined as 50%–70% of the age-predicted maximum heart rate, calculated using the formula: 220 minus age (Hidalgo and Sotos, 2021).

#### 2.4.2 Traditional cognitive training (TCT)

Current clinical approaches to TCT for MCI employ heterogeneous protocols, with significant variations in training modalities (Hill et al., 2017) (computerized vs. paper-and-pencil vs. VR), cognitive domains targeted (Sherman et al., 2020) (single-domain vs. multi-domain approaches), intervention parameters (Ren et al., 2024) (session duration, frequency, and total training dose) and theoretical frameworks (Park and Bischof, 2013) (strategy-based vs. process-specific vs. neuroplasticity-focused). Based on our preliminary survey and the level of acceptance among older adults in the community, the TCT program was designed around classical cognitive domains commonly used in cognitive neuroscience, incorporating Card-based Visual Recognition, Spot the Difference, Jigsaw Puzzles, and Sudoku (Table 2). The training targets four key cognitive domains: memory, attention, executive function, and visuospatial ability. After each physical exercise session, the intervention group will complete TCT (15 min per session, three times per week for 12 weeks), with one task randomly selected for each session.

**TABLE 2:** Traditional cognitive training (TCT) and virtual reality cognitive training (VRCT) programs.

Groups	Domains	Programs	Instructions
TCT			
	Memory	Card-based visual recognition task	The therapist prepares picture cards featuring items such as fruits, animals, and vehicles. The participant is first shown each picture one by one. After viewing, the pictures are covered, and the participant is asked to recall and verbally name the items that were shown.
	Attention	Spot the difference task	Place two similar pictures on the table. The participant is asked to pick up the images, identify the differences between them, and mark the areas where the differences are found.
	Visuospatial ability Executive function	Jigsaw puzzles	The participant is presented with puzzle pieces of a chosen image (e.g., nature scenes, animals, or familiar objects). They are asked to observe the shapes, colors, and edges, and gradually fit the pieces together. Puzzle difficulty can be adjusted by changing the number of pieces, complexity of the image, or removing edge pieces.
	Visuospatial ability Executive function	Sudoku	Sudoku chessboard consists of a 9 × 9 grid divided into nine 3 × 3 subgrids. The objective is to fill the grid so that each row, each column, and each 3 × 3 box contains the digits from 1 to 9 without repetition.
VRCT			
	Memory Visuospatial ability Executive function	Memory cards	Various colored pattern cards will appear on the screen, and participants must memorize their shapes and positions within a set time. Once the countdown ends, the cards will flip over, and participants are required to select the correct color or pattern based on the task instructions.
	Attention Visuospatial ability Executive function	Letter-number sequencing	Scattered sequences of numbers or letters will appear in a 3D virtual space. Participants must observe and analyze them carefully, then arrange them according to specific rules.
	Memory Attention Executive function	Butterfly memory	Participants observe butterflies flying across the screen and must remember their quantity, color, and flight order, then select the correct answer according to the task instructions.
	Memory Visuospatial ability Executive function	Supermarket shopping	This training simulates a real-life shopping scenario. Participants read the task instructions, memorize the types and quantities of items to be purchased, and then select the required items from memory.
	Mind-body relaxation	Mindfulness meditation	The system offers 16 VR scenarios designed for mindfulness-based relaxation. Guided by the system’s instructions, participants are led to focus on the present moment through breathing or visualization, promoting a state of physical and mental relaxation.

TCT, traditional cognitive training; VRCT, virtual reality cognitive training.

#### 2.4.3 Virtual reality cognitive training (VRCT)

To enhance participant engagement and compliance, we added the VRCT program. VRCT is delivered using the SY-VRD VR system (Shuyun, Chengdu, China). Drawing on preliminary research and participant feedback, we selected four game-based cognitive tasks—Memory Cards, Letter-Number Sequencing, Butterfly Memory, and Supermarket Shopping—designed to target attention, memory, visuospatial skills, and executive function, with each task engaging multiple cognitive domains (Kim et al., 2023; Ren et al., 2024; Table 2). The SY-VRD system incorporates an adaptive algorithm that automatically adjusts task difficulty based on individual performance. Detailed operational procedures and difficulty parameters for each task are provided in the Supplementary materials. Additionally, to optimize intervention adherence and mitigate potential VR-related fatigue, each VRCT session concluded with a 5-min guided mind-body relaxation protocol. A professional therapist is available to guide participants in using the equipment and to offer support as needed; however, all tasks are completed independently by the participants. The intervention group will complete a 15-min VRCT session following each TCT session, with one VRCT task randomly selected for each session.

### 2.5 Outcome measurements

The primary outcome is the change from baseline in a memory-weighted cognitive composite score, specifically designed to detect subtle changes over the trial period. This composite integrates tests that assess the cognitive domains most relevant to MCI, including: (1) Verbal learning and memory: total of five learning trials from the Chinese Auditory Verbal Learning Test (AVLT); (2) Delayed recall: 20-min delayed recall trial from the Chinese AVLT; (3) Executive function: Trail Making Test (TMT; Parts A and B); (4) Attention and working memory: Digit Span Test (DST; forward and backward).

To construct the composite score, raw scores from each test will be converted to Z-scores using the mean and standard deviation of the pooled sample at each test time point. For the Trail Making Test, where lower scores indicate better performance, Z-scores will be multiplied by −1 to ensure consistent directionality across all tests (higher Z-scores always indicate better performance). Based on the theoretical importance of memory function in MCI, the AVLT learning and delayed recall scores will receive a weight of 2, while the TMT and DST scores will receive a weight of 1. The final composite score will be calculated as the weighted average of these Z-scores (Andrade, 2021).

Secondary cognitive outcomes include changes from baseline in global cognitive function, assessed using the Montreal Cognitive Assessment (MoCA) and the Mini-Mental State Examination (MMSE). The MoCA provides a comprehensive assessment of multiple cognitive domains with high sensitivity for MCI (Nasreddine et al., 2005), while the MMSE is included to facilitate comparison with prior studies. Subjective cognitive decline will be assessed using the Alzheimer’s Disease 8-item scale (AD8), an informant-based measure that captures perceived changes in memory, orientation, and executive function.

Exploratory outcomes include the following: (1) Changes in gut microbiota alpha/beta diversity and composition assessed by 16S rRNA gene sequencing, (2) Changes in resting-state brain activity and functional connectivity assessed by fMRI. (3) changes in serum metabolites, evaluated via metabolomic analysis; (4) changes in sleep quality, measured by the Insomnia Severity Index (ISI); (5) changes in mood, assessed using the Patient Health Questionnaire-9 (PHQ-9) and the Generalized Anxiety Disorder-7 (GAD-7) scales; (6) changes in daily living abilities, measured by the Instrumental Activities of Daily Living (IADL) and Activities of Daily Living (ADL) scales; (7) changes in gastrointestinal symptoms, assessed using the Gastrointestinal Symptom Rating Scale (GSRS); (8) changes in quality of life, measured by the WHO Quality of Life-OLD (WHOQOL-OLD) scale.

Safety evaluation will include the incidence and severity of all adverse events (AEs), with particular attention to VR-related discomfort (e.g., dizziness, nausea, eyestrain, headache). AEs will be systematically recorded after each training session, and vital signs (blood pressure, heart rate) will be monitored immediately post-session. All AEs will be graded as mild, moderate, or severe based on participant self-report.

### 2.6 Biological data acquisition

Biological data will be collected at three time points: baseline, trial completion (week 12), and 12-week follow-up (week 24). MRI scans will be acquired using a 3.0T Siemens Skyra scanner (Erlangen, Germany) at Zigong Mental Health Center. The data to be analyzed will include gray matter volume, cortical thickness, amplitude of low-frequency fluctuations (ALFF), bilateral hippocampal connectivity, posterior cingulate cortex (PCC) functional connectivity, default mode network (DMN) connectivity, and frontal network connectivity. Additionally, blood samples will undergo metabolomic analysis using mass spectrometry, while fecal samples will be analyzed for microbiome changes via 16S rRNA gene sequencing. All laboratory procedures will follow standardized protocols.

### 2.7 Procedure

**FIGURE 1:**
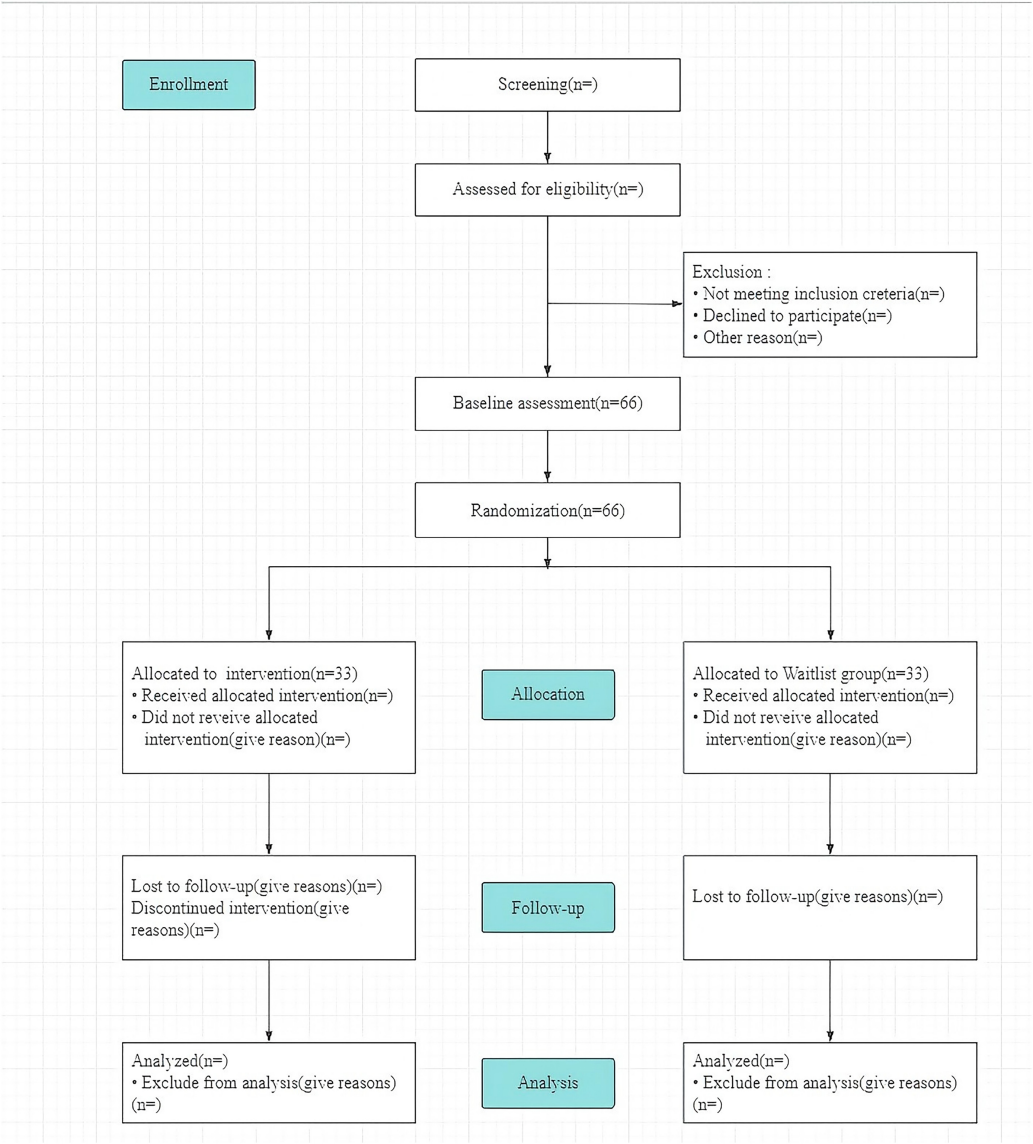
Consolidated Standards of Reporting Trials (CONSORT) diagram.

Participants are being recruited through community health center flyers and digital advertisemts onen WeChat platform. Interested individuals attend screening visits at local community health centers where they complete standardized assessments including the AD8, ISI, PHQ-9, GAD-7, MMSE, and MoCA scales. Potentially eligible individuals will undergo face-to-face diagnostic evaluations conducted by senior psychiatrists from the Zigong Mental Health Center. Participants diagnosed with MCI who meet inclusion and exclusion criteria undergo informed consent procedures followed by comprehensive baseline assessments within 1 week, including collection of demographic data, 3-day food diary, functional assessments (ADL and IADL), symptom and quality-of-life measures (GSRS and WHOQOL-OLD), and biological data (blood samples, fecal samples, cranial MRI). Participants are then randomly allocated to either the intervention group or the waitlist group. The intervention group will receive a combination of PE, TCT, and VRCT, while the control group will not receive any intervention during the study period. To compensate, the waitlist control group will be offered the same training after the follow-up assessment. The CONSORT diagram depicting participant flow is presented in Figure 1, and the time points for data collection are detailed in Table 3.

**TABLE 3:** Outline of study assessments and timelines.

Items	Screening	Week 0 (baseline)	Week 6 (mid-intervention)	Week12 (post-intervention)	Week 24 (12-week follow-up)
Informed consent	–	×	–	–	–
Randomization	–	×	–	–	–
Demographic information	–	×	–	–	–
Food diary	–	×	–	×	×
AD8	×	–	×	×	×
ISI	×	–	×	×	×
PHQ9	×	–	×	×	×
GAD7	×	–	×	×	×
AVLT	–	×	×	×	×
TMT (A and B)	–	×	×	×	×
DST (forward and backward)	–	×	×	×	×
MMSE	×	–	×	×	×
MOCA	×	–	×	×	×
ADL	–	×	×	×	×
IADL	–	×	×	×	×
GSRS	–	×	×	×	×
WHOQOL-OLD	–	×	×	×	×
Blood sample collection	–	×	–	×	×
Fecal sample collection	–	×	–	×	×
Magnetic resonance imaging (MRI)	–	×	–	×	×

AD8, Alzheimer’s Disease 8-item scale; ISI, Insomnia Severity Index; PHQ-9, Patient Health Questionnaire-9; GAD-7, Generalized Anxiety Disorder 7-item scale; MMSE, Mini-Mental State Examination; MOCA, Montreal Cognitive Assessment; ADL, Activities of Daily Living scale; IADLs, Instrumental Activities of Daily Living scale; GSRS, Gastrointestinal Symptom Rating Scale; WHOQOL-OLD, World Health Organization Quality of Life Instrument–Older Adults Module; AVLT, Chinese Auditory Verbal Learning Test; TMT (A and B), Trail Making Test (Parts A and B); DST, Digit Span Test (forward and backward).

### 2.8 Statistical analysis

#### 2.8.1 Sample size

Sample size estimation was performed using G*Power 3.1.9.7 based on the primary outcome of change in the memory-weighted cognitive composite score. Assuming a moderate effect size (f = 0.25), α = 0.05, and 90% power for a repeated-measures ANOVA, the minimum required sample size is 46 participants. To account for a 30% dropout rate, we will enroll 66 participants.

#### 2.8.2 Statistical considerations

The analysis will adhere to the eHealth Consolidated Standards of Reporting Trials (CONSORT) criteria (Eysenbach, 2011), follow the intention-to-treat principle and per protocol set. The per-protocol set included participants who attended at least 70% of the assigned intervention sessions and completed all protocol-specified assessments (Zhang et al., 2018). Statistical analyses will be conducted using IBM SPSS Statistics (Version 24.0; IBM Corp., Armonk, NY, United States). Continuous variables are presented as mean and standard deviation; categorical variables as frequency and percentages.

For baseline characteristics, continuous variables are compared between groups using *t*-test, while categorical variables are analyzed using chi-square test. Primary outcomes will be analyzed using two-way repeated-measures ANOVA, evaluating the main effects of group, time, and their interaction. *Post-hoc* pairwise comparisons were conducted with Bonferroni correction for multiple comparisons. Missing follow-up data will be imputed using the last observation carried forward (LOCF) method. Sensitivity analyses (completers-only and per-protocol) will assess potential bias due to dropouts and imputation methods. We will also compare baseline characteristics between participants who dropped out and those who completed the study. All tests are two-sided, with statistical significance defined as *p* < 0.05.

Multimodal neurobiological correlates of treatment response will be assessed by integrating structural/functional MRI, gut microbiome, and plasma metabolome with behavioral outcomes. For analyses involving gut microbiota, key dietary patterns extracted from the 3-day food diaries will be included as covariates to enhance the validity of the findings. Hypothesis-driven analyses will include: (i) partial least squares (PLS) regression to identify multivariate brain-behavior associations, (ii) mixed-effects modeling for longitudinal effects, and (iii) mediation analysis to test whether brain-gut axis changes drive clinical improvement. Significance thresholds will be adjusted via Holm-Bonferroni correction.

#### 2.8.3 Safety analysis

Safety considerations will be a priority throughout the study. All adverse events (AEs) will be carefully documented. AEs are defined as any unfavorable or unintended sign, symptom, or disease temporally associated with the study intervention. Special attention will be paid to VR-related discomfort (e.g., dizziness, nausea, eyestrain, and headache). AEs will be systematically queried and recorded immediately after each training session. Vital signs (blood pressure and heart rate) will be monitored immediately after each training session. All AEs will be graded for severity based on participant self-report, using the following criteria: Mild (easily tolerated), Moderate (interferes with usual activities), or Severe (incapacitating). To comprehensively evaluate the safety profile, the incidence and severity distribution of adverse events will be analyzed using descriptive statistics for all randomized participants.

## 3 Discussion

This study aims to (1) evaluate the efficacy of adjunctive VRCT in improving cognitive function in MCI, and (2) investigate potential brain-gut axis mechanisms mediating intervention effects through multimodal biomarker analyses.

Virtual reality cognitive training, as a demand-adapted cognitive training approach, offers an effective alternative that addresses several limitations of TCT, such as its time-consuming and labor-intensive nature (De Luca et al., 2020). Previous studies have suggested that VRCT may help delay or prevent dementia in high-risk middle-aged populations (Doniger et al., 2018). This effect is thought to stem from its capacity to enhance cognitive reserve and promote neuroplasticity (Teo et al., 2016). Furthermore, VRCT provides immersive and interactive experiences by simulating real-world environments, effectively integrating cognitive and physical training components (Stone et al., 2011). We hypothesize that this unique feature may compensate for the limitations of TCT and enhance its efficacy.

Recent evidences suggest that cognitive impairment is associated with specific gut microbial communities (Aljumaah et al., 2022; Kesika et al., 2021; Tooley, 2020). This finding suggests that cognitive decline may be improved through interventions such as probiotics, prebiotics, and commensal bacteria. Indeed, a recent meta-analysis indicates that probiotic supplementation may enhance cognitive function, particularly among individuals with MCI (Zhu et al., 2021). Moreover, brain imaging studies have provided evidence that gut microbiota are associated with alterations in brain structure and function (Tooley, 2020), further supporting their relationship with cognition. However, although cognitive training has been shown to improve cognition, few studies have explored its role in influencing the brain-gut axis. We hypothesize that adjunctive VRCT may mediate cognitive improvement in patients with MCI by influencing gut microbial composition, metabolic profiles, and brain function and structure. However, this hypothesis remains inconclusive. To explore potential neurobiological associations, we will collect multimodal data, including MRI, serum metabolomics, gut microbiomics, and comprehensive cognitive assessments.

This study has several limitations. First, the study population comprised individuals with MCI, a highly heterogeneous group that may progress to diverse dementia subtypes over extended observational periods. Given the time-limited nature of our cognitive training intervention, we were unable to evaluate whether the combined intervention delays dementia onset. Second, this is a single-blinded study, so there may have been a placebo effect interfering with the experimental results. However, we will blind the raters and statisticians to minimize potential bias. Third, our study population consisted of older adults, and the potential presence of comorbid conditions may have influenced the experimental outcomes. We will analyze the impact of these comorbidities as potential confounding factors in our results. Fourth, It is important to acknowledge that the multi-modal nature of our intervention introduces potential non-specific effects. The intervention group received enhanced social interaction, professional guidance, and exposure to novel technology, which may have contributed to the observed outcomes through psychosocial mechanisms such as the placebo effect or Hawthorne effect. While these elements are integral to the real-world delivery of such an intervention, future studies with dismantling designs could help isolate the specific contribution of each component. Fifth, we adopted square dancing as the form of physical exercise primarily to enhance participants’ engagement and adherence, although individual variations in exercise intensity may exist. Moreover, square dancing is deeply rooted in specific cultural and regional contexts, which may hinder its adoption and effectiveness in other geographical or cultural settings. Sixth, although the MoCA was initially registered as the primary outcome, we subsequently adopted a memory-weighted cognitive composite as the revised primary outcome to provide greater sensitivity for detecting subtle cognitive changes over the 24-week trial period. Finally, some elderly participants may experience discomfort when wearing virtual reality equipment for extended periods. To address this, we intentionally incorporated a mind-body relaxation section to help reduce tension and improve comfort.

In summary, this study investigates the efficacy of adjunctive VRCT in individuals with MCI, along with its underlying brain-gut axis mechanisms. If effective, this combined VRCT approach may offer an accessible, non-pharmacological strategy for preventing dementia progression in community-dwelling older adults. Our findings may contribute novel insights to this emerging field and inform the development of innovative therapeutic strategies to slow dementia progression.
