# Supplementary material for: A novel virtual reality-integrated multi-modal intervention for community-dwelling older adults with mild cognitive impairment: protocol for a randomized controlled trial
Source: Front Aging Neurosci. 2026 Mar 5;18:1721346. doi: 10.3389/fnagi.2026.1721346 (PMC12999948; doi:10.3389/fnagi.2026.1721346)
Supplement: Supplementary file 1 [file Table_1.docx]

**Virtual Reality Cognitive Training (VRCT) Intervention Manual**

1. System Overview

The VRCT program is delivered using the SY-VRD system. The intervention includes four cognitive training tasks and one relaxation exercise. Each session lasts 15 minutes, consisting of one randomly selected cognitive task followed by 5 minutes of mindfulness meditation.

2. General Adaptive Difficulty Rules

Task difficulty follows an adaptive algorithm based on participant performance. Difficulty increases after achieving >80% accuracy in consecutive sessions and decreases if accuracy falls below 50%.

3. Task Instructions and Difficulty Parameters

3.1 Memory Cards

Instructions: Participants memorize the positions and patterns of cards displayed on a grid within a set time, then match them after they are hidden.

Difficulty Parameters:

Level 1: 6 cards (3 pairs), 10-second memorization

Level 2: 8 cards (4 pairs), 8-second memorization

Level 3: 12 cards (6 pairs), 6-second memorization

3.2 Letter-Number Sequencing

Instructions: Participants observe numbers and letters in a 3D space and sequentially select them in ascending order (numbers first, then letters).

Difficulty Parameters:

Level 1: 3-item sequence (e.g., 1, A, 2)

Level 2: 5-item sequence (e.g., 1, A, 2, B, 3)

Level 3: 7-item sequence (e.g., 1, A, 2, B, 3, C, 4)

3.3 Butterfly Memory

Instructions: Participants recall the quantity, color, and order of butterflies flying across the screen.

Difficulty Parameters:

Level 1: 4 butterflies, 2 colors, immediate recall

Level 2: 6 butterflies, 3 colors, 3-second delay

Level 3: 8 butterflies, 4 colors, 5-second delay

3.4 Supermarket Shopping

Instructions: Participants memorize a shopping list, navigate a virtual supermarket, select items, and stay within a budget.

Difficulty Parameters:

Level 1: 3 specific items (e.g., milk, bread, apples), no budget limit

Level 2: 5 items, including categorical items (e.g., "a fruit"), lenient budget

Level 3: 7 items with specific attributes and strict budget constraints

3.5 Mindfulness Meditation

Instructions: Participants engage in guided mindfulness relaxation across 16 immersive VR environments.

Parameters: Non-adaptive; environment selection is participant-driven to support engagement and adherence.

4. Data and Safety Monitoring

System records include task accuracy, reaction time, and difficulty progression. Adverse events (e.g., dizziness, nausea) are documented post-session using a standardized severity scale (Mild/Moderate/Severe).
